# Online adaptive radiotherapy compared to plan selection for rectal cancer: quantifying the benefit

**DOI:** 10.1186/s13014-020-01597-1

**Published:** 2020-07-09

**Authors:** R. de Jong, K. F. Crama, J. Visser, N. van Wieringen, J. Wiersma, E. D. Geijsen, A. Bel

**Affiliations:** Department of Radiation Oncology, Amsterdam UMC, University of Amsterdam, Meibergdreef 9, 1105AZ, Amsterdam, The Netherlands

**Keywords:** Adaptive radiotherapy, Adaptive treatment, Rectal cancer, Plan selection, Library of plans, Plan of the day, Normal tissue sparing

## Abstract

**Background:**

To compare online adaptive radiation therapy (ART) to a clinically implemented plan selection strategy (PS) with respect to dose to the organs at risk (OAR) for rectal cancer.

**Methods:**

The first 20 patients treated with PS between May–September 2016 were included. This resulted in 10 short (SCRT) and 10 long (LCRT) course radiotherapy treatment schedules with a total of 300 Conebeam CT scans (CBCT). New dual arc VMAT plans were generated using auto-planning for both the online ART and PS strategy.

For each fraction bowel bag, bladder and mesorectum were delineated on daily Conebeam CTs. The dose distribution planned was used to calculate daily DVHs. Coverage of the CTV was calculated, as defined by the dose received by 99% of the CTV volume (D99%). The volume of normal tissue irradiated with 95% of the prescribed fraction dose was calculated by calculating the volume receiving 95% of the prescribed fraction or more dose minus the volume of the CTV. For each fraction the difference between the plan selection and online adaptive strategy of each DVH parameter was calculated, as well as the average difference per patient.

**Results:**

Target coverage remained the same for online ART. The median volume of the normal tissue irradiated with 95% of the prescribed dose dropped from 642 cm3 (PS) to 237 cm3 (online-ART)(*p* < 0.001). Online ART reduced dose to the OARs for all tested dose levels for SCRT and LCRT (*p* < 0.001). For V15Gy of the bowel bag the median difference over all fractions of all patients was − 126 cm^3^ in LCRT, while the average difference per patient ranged from − 206 cm^3^ to − 40 cm^3^. For SCRT the median difference was − 62 cm^3^, while the range of the average difference per patient was − 105 cm3 to − 51 cm^3^.

For V15Gy of the bladder the median difference over all fractions of all patients was 26% in LCRT, while the average difference per patient ranged from − 34 to 12%. For SCRT the median difference of V95% was − 8%, while the range of the average difference per patient was − 29 to 0%.

**Conclusions:**

Online ART for rectal cancer reduces dose the OARs significantly compared to a clinically implemented plan selection strategy, without compromising target coverage.

**Trial registration:**

Medical Research Involving Human Subjects Act (WMO) does not apply to this study and was retrospectively approved by the Medical Ethics review Committee of the Academic Medical Center (W19_357 # 19.420; Amsterdam University Medical Centers, Location Academic Medical Center, Amsterdam, The Netherlands).

## Background

Pre-operative external beam radiotherapy combined with chemotherapy and followed by surgery is standard of care for non-metastasized locally advanced rectal cancer [1]. For any treatment site in the pelvic region, radiotherapy is associated with toxicity due to the inevitable dose to the organs at risk (OAR) such as the bladder and small bowel [2]. Shaping the dose with steep dose gradients using Intensity Modulated Radiation therapy (IMRT) or Volumetric Modulated Arc Therapy (VMAT) has become common practice [3, 4], but to take optimal advantage of these treatment techniques, adequate PTV margins and visualization of the target volume prior to each treatment fraction to avoid misses is essential. Population-based margins are typically very large in the pelvic region due to large day-to-day variations of the target volume [5–9]. Online Conebeam CT (CBCT) image guidance at the treatment machine is widely applied and very effective in this patient group for verification of patient position and target coverage, while using the population-based (fixed) margins.

Even with its limited soft-tissue contrast online CBCT image guidance can also be used for an adaptive procedure using plan selection. Plan selection using more individualized and therefore smaller margins enables reducing the dose to OARs and has proven its value in the treatment of bladder and cervix [10–14]. For rectal cancer we analyzed previously in a simulated study [15, 16] and prospective clinical study [17] such a plan selection strategy yields only a small advantage for the average population but has a benefit for individual patients and has been clinical practice in our department since 2016. Recent developments in improved image quality for treatment guidance, such as MRI-guided radiotherapy and CBCT guidance, as well as developments in fast and precise auto-contouring [18] and auto-planning ‘marks the beginning of a new era’ [19]. Online adaptive treatment, based on both MRI and CBCT guidance, is now a real possibility [19–22] and surely promising in reducing dose to the OARs even further.

Although the first step towards individualized margins using plan selection has been proven feasible and is clinically implemented for rectal cancer [16, 23], daily online adaptation is expected to be beneficial. To our knowledge the benefit of online adaptation for rectal cancer has not been reported yet, with a clinically implemented plan selection as the baseline.

The aim of this study is to assess the added value of online adaptive radiotherapy (online ART) for rectal cancer, by comparing the online adaptive treatment to a clinically implemented plan selection strategy by quantifying the benefit with respect to dose to the OARs and coverage of the target volume.

## Methods

In this study we used the same methodology and patient cohort [17] as in our previous study, a comparison between a fixed margin and a variable margin technique, e.g. plan selection, but applied to a clinical cohort of LCRT (25x2Gy) and SCRT (5x5Gy). For the plan selection distribution there is a brief summary (Supplement 1, 2) as the results are described in our previous article [17].

### Patients

The first twenty consecutive patients that underwent plan selection between May and September 2016 were included in this study. This cohort included 20 patients of which 10 patients were treated with a short course treatment (5x5Gy)(SCRT) and 10 patients with a long course treatment (25x2Gy)(LCRT). Boost dose to the tumor is not part of the treatment regimen. Patient characteristics can be found in Supplement 3. The schedule regimen was determined by stage and resectability of the primary tumor. All fractions (*N* = 300) were used for analysis. All patients were positioned supine with a knee support and their arms raised over their heads (Posirest, CIVCO).

### Planning CT and target volume

A planning CT was acquired with a full bladder, patients were instructed to empty the bladder 1.5 h before CT acquisition and to drink subsequently 0.5 l of fluid. No additional instructions have been given with respect to rectal filling and thus, spontaneous rectum filling was used.

Structures, based on the delineation guidelines by Roels et al. [24], were contoured using Advantage SIM (GE) or Velocity (Velocity, AI 3.2, Varian Medical Systems).

The GTV, defined as tumor and positive lymph nodes, was delineated. The tumor itself is indicated on the reference scan but no boost dose is applied to the tumor. For CTV, the mesorectum, presacral space, internal iliac lymph node regions and, when applicable, obturator lymph node regions, were delineated by a radiation oncologist. With the transition at the base of the bladder the mesorectum was divided into an upper and lower part to be able to differentiate margins between the upper and lower mesorectum based on the geometrical uncertainties reported by Nijkamp et al. [6, 7, 9]. Radiation Therapists (RTTs) contoured the OARs (i.e., the bladder, bowel bag for small bowel and femur heads) according to RTOG guidelines [25].

### Plan selection margins

PTV margins for the clinically applied plan selection strategy around the CTV lymph node regions were created by expanding the volumes with 8 mm in all directions. The CTV pre sacral space was expanded with 10 mm in all directions. For the upper and lower mesorectum the volume was expanded with 10 mm in all directions, except for the ventral side. The ventral side of the lower mesorectum had an fixed anterior margin of 15 mm, whereas the ventral side of the upper mesorectum had variable anterior margins to use for plan selection. Two sets of margins were defined according to the anatomy captured on the planning CT scan: For an empty rectum (Supplement 4(a)) on planning CT we used PTV margins of 25 mm, 15 mm, 0 mm, as − 15 mm was unlikely to be needed. For a full rectum on planning CT (Supplement 4(b)), we used 15 mm, 0 mm and − 15 mm, as 25 mm was unlikely to be needed [15, 16].

### Clinical procedure – registration, correction and plan selection

All CBCT scans were registered to the pelvic bony anatomy (XVI5.0, Elekta) using translations and rotations. If the rotation around one of the axes was larger than 4°, the patient was re-aligned. Remaining setup rotations under 4° were converted into a table correction (translations-only) by taken out the rotations using a rotation point at the center of gravity of the PTV. This means that rotational errors were still present during treatment delivery. Based on the anatomy of the day the smallest plan encompassing the target volume was selected to treat the patient.

### Delineations

A graphic overview of the workflow can be found in Supplement 5. Each CBCT scan (*N* = 300) was resampled to the planning CT including the online table correction, which represented the anatomy of the patient at treatment, and exported to Velocity. For this study, delineations used in our previous study on these CBCT scans were available of upper and lower mesorectum as well as bladder and bowel bag. These structures were delineated by a single experienced observer (RdJ). The elective lymph nodes and pre sacral space were not re-delineated. Instead, the delineations were propagated from the planning CT to the CBCT using a bony anatomy match. The total CTV volume was uniformly expanded with 3 mm to create the PTV for the online adaptive strategy. Using the identity transformation, these structures were then propagated to the planning CT scan.

### Dose calculation and comparison between the online adaptive treatment and plan selection strategy

For the plan selection strategy a new plan library was created and for the online adaptive strategy a treatment plan was created for each fraction, in both cases using automated planning [26] with the same clinical goals with the same prioritization (Plan Explorer, RaySearch v6.99). The planning technique was VMAT dual arc with energy 10 MV using the planning CT for dose calculation. In order to avoid treatments plans with too much modulation, for each arc a maximum of 300 MU and 750 MU was allowed for plans with 2 Gy and 5 Gy prescribed fraction dose, respectively. All plans were checked for clinical acceptability. This resulted in 300 treatment plans for the online adaptive treatment and 60 plans for the plan selection strategy.

### Evaluation

For both the plan selection strategy and the online adaptive strategy the DVHs for each fraction were calculated using the planned dose distribution on the planning CT together with the delineated structures from the registered CBCT. For the plan selection strategy only the selected plan for that fraction was used. Consequently only the anatomical changes to structures delineated on CBCT were taken into account. Literature on predictive dose volume parameters is relatively sparse, therefore a range of DVH parameters based on the parameters suggested in de QUANTEC papers [27, 28] as well as DVH parameters suggested by Mouttet-Audouard et al. [29] and Deville et al. [30, 31] were evaluated (i.e. the volume receiving at least 15 Gy (V15Gy), 30 Gy (V30Gy), 40 Gy (V40Gy), 45 Gy (V45Gy)). Deformable registration inevitably involves uncertainties [32], especially in the pelvic region with e.g. appearing and disappearing gas. For the comparison between the online adaptive and variable margin technique, e.g. plan selection, we chose to avoid dose accumulation by deformable registration for assessing the total dose to OARs. Instead the corresponding fractional dose levels were used and the difference of the dose to the OARs between the online adaptive treatment and plan selection strategy was tested *per fraction*.

The fractional dose levels analyzed for LCRT were V0.6Gy (equals V15Gy), V1.2Gy (equals V30Gy), V1.6Gy (equals V40Gy), V1.8Gy (equals V45Gy), V95%. The fractional dose levels analyzed for SCRT were V3.0Gy (equals V15Gy), V95%. Dose levels higher than the prescribed dose were skipped from evaluation. The mean dose (Dmean) for bladder was analyzed for both SCRT and LCRT.

For each fraction the difference between the plan selection and online adaptive strategy of each DVH parameter was calculated, as well as the average difference per patient.

We calculated target coverage as a percentage of the prescribed dose received by 99% of the CTV volume (D99%).

### Statistical analysis

Wilcoxon signed-rank sum tests were used to test the difference between online adaptive strategy and plan selection for:

1. Volume of normal tissue irradiated with 95% of the prescribed fraction dose defined by the volume receiving 95% of the prescribed dose or more minus the volume of the CTV.
2. Difference of all DVH parameters for dose to the OARs per fraction.

Significance was set at *p* < 0.05. Statistical analysis was performed using SPSS25.

## Results

Target coverage for the total cohort, expressed as D99%, the percentage of the prescribed dose received by 99% of the CTV volume, was on average 98.5% for plan selection and 98.7% for online adaptive strategy.

### 1) Volume of normal tissue irradiated with 95% of the prescribed dose

**Fig. 1:**
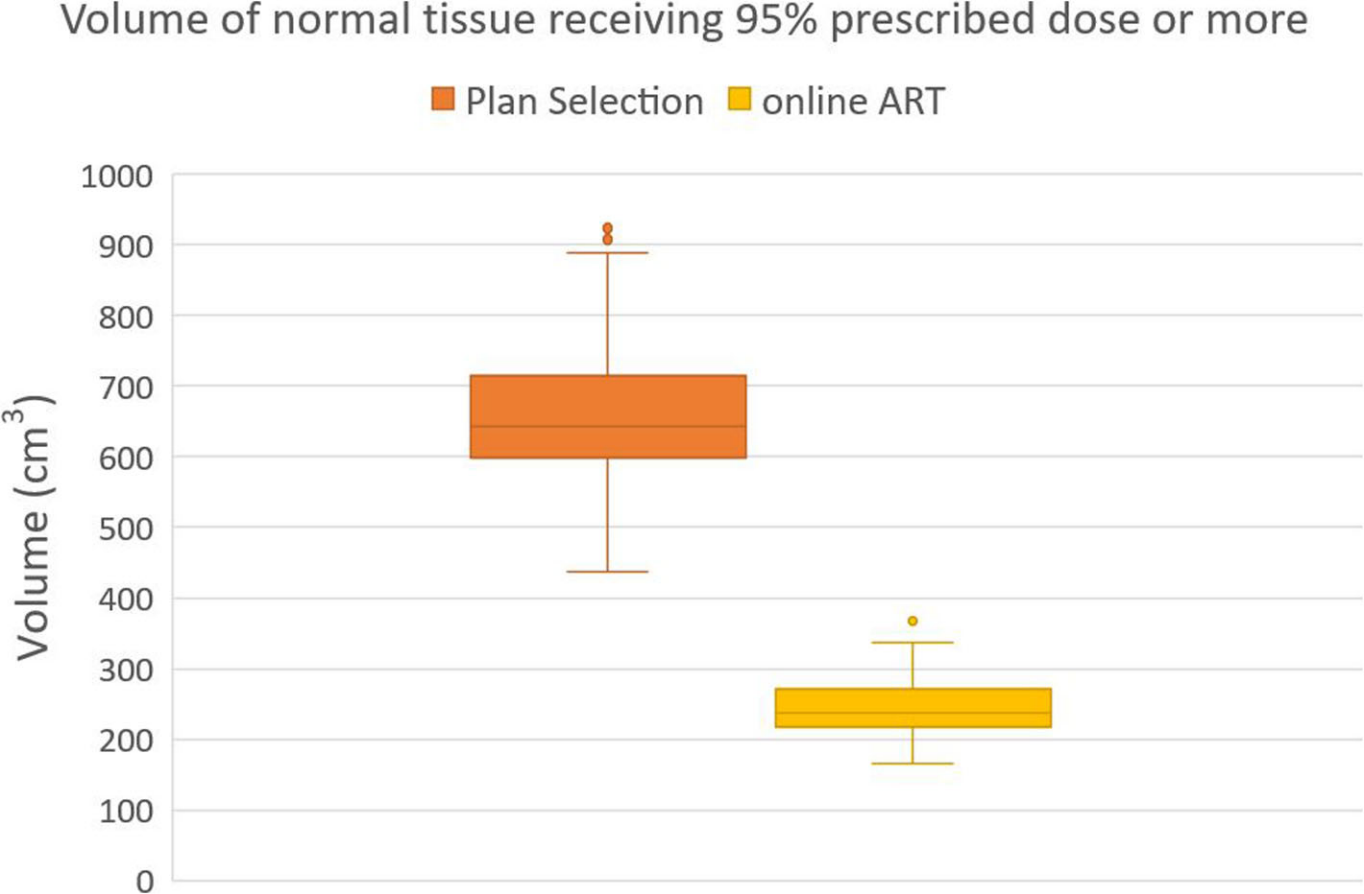
Boxplot showing difference in normal tissue irradiated between Plan selection and Online ART for the total cohort. The boxplot shows the interquartile range. Whiskers indicate the 5th and 95th percentiles. Outliers (°) are marked

For the total cohort the median volume of the normal tissue irradiated with 95% of the prescribed dose dropped from 642 cm^3^ using plan selection to 237 cm^3^ using the online adaptive strategy, which was statistically significant (*p* < 0.001) (Fig. 1).

### 2) Dose to the organs at risk

**Fig. 2:**
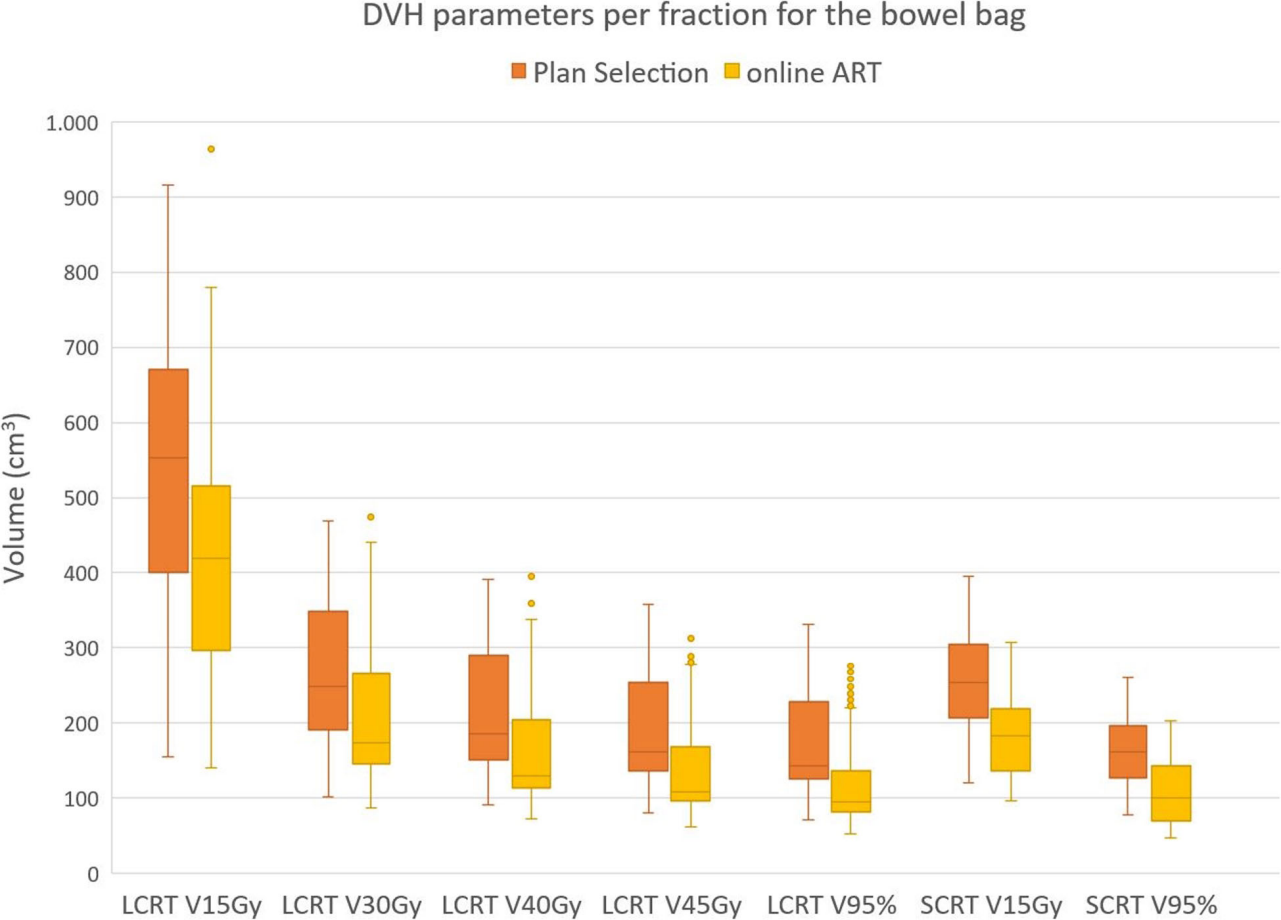
Boxplot showing the volume of small bowel receiving x Gy for different DVH parameters for both Plan selection and online ART. The boxplot shows the interquartile range. Whiskers indicate the 5th and 95th percentiles. Outliers (°) are marked

**Fig. 3:**
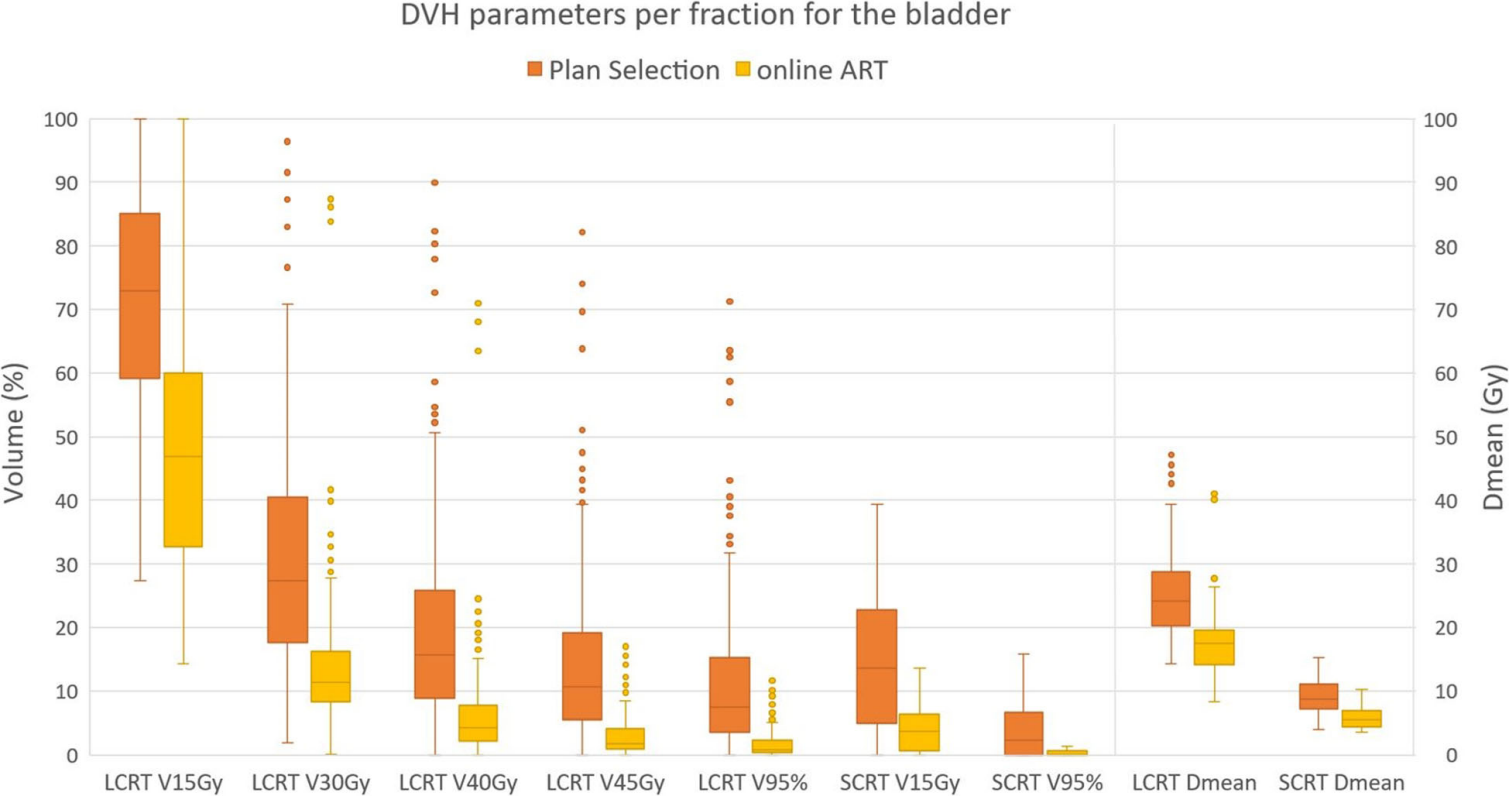
Boxplot showing the volume of bladder receiving x Gy for different DVH parameters for both Plan selection and online ART. The boxplot shows the interquartile range. Whiskers indicate the 5th and 95th percentiles. Outliers (°) are marked

Overall, compared to the plan selection strategy, the online adaptive strategy reduced dose to the bowel bag (*p* < 0.001) (Fig. 2) and bladder (*p* < 0.001) (Fig. 3) for all dose levels.

**Fig. 4:**
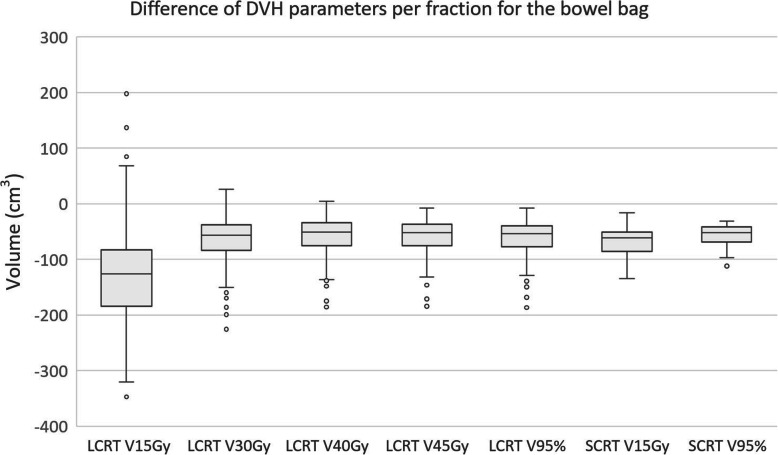
Boxplot showing the difference in volume of small bowel receiving x Gy for different DVH parameters. The boxplot shows the interquartile range. Whiskers indicate the 5th and 95th percentiles. Outliers (°) are marked

For V15Gy of the bowel bag the median difference over all fractions of all patients was − 126 cm^3^ in LCRT, while the average difference per patient ranged from − 206 cm^3^ to − 40 cm^3^. For SCRT the median difference was − 62 cm^3^, while the range of the average difference per patient was − 105 cm^3^ to − 51 cm3. Boxplots of the differences of all DVH parameters are shown in Fig. 4, while the range of the average differences per patient can be found in Table 1.

**Table 1:** Average and range of the average differences per DVH parameter per patient

				AVG	min	max
Bowel bag	LCRT	V15	(cm^3^)	−127	−206	−40
	LCRT	V30	(cm^3^)	−64	− 117	−19
	LCRT	V40	(cm^3^)	−58	−102	−25
	LCRT	V45	(cm^3^)	−60	−101	−30
	LCRT	V95%	(cm^3^)	−62	−101	−34
	SCRT	V15	(cm^3^)	−69	−105	−51
	SCRT	V95%	(cm^3^)	−57	−86	−42
Bladder	LCRT	V15	(%)	−24	−34	12
	LCRT	V30	(%)	−18	−39	−3
	LCRT	V40	(%)	−14	− 39	−4
	LCRT	V45	(%)	−12	−36	− 3
	LCRT	V95%	(%)	−10	−32	−2
	LCRT	Dmean	Gy	−8	−15	0
	SCRT	V15	(%)	−11	−29	0
	SCRT	V95%	(%)	−4	−12	0
	SCRT	Dmean	Gy	−3	−8	0

**Fig. 5:**
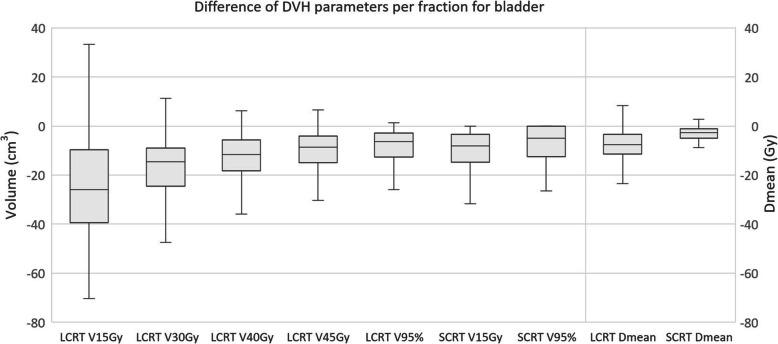
Boxplot showing the difference in volume of bladder receiving x Gy for different DVH parameters. The boxplot shows the interquartile range. Whiskers indicate the 5th and 95th percentiles. Outliers (°) are marked

For V15Gy of the bladder the median difference over all fractions of all patients was 26% in LCRT, while the average difference per patient ranged from − 34 to 12%. For SCRT the median difference of V95% was − 8%, while the range of the average difference per patient was − 29 to 0%. Boxplots of the differences of all DVH parameters are shown in Fig. 5, while the range of the average differences per patient can be found in Table 1.

## Discussion

This study is the next step in our research to improve radiotherapy treatment for rectal cancer. After reporting on the possible benefit with a simulated plan selection strategy for SCRT [15, 16] and a prospective comparison for both SCRT and LCRT [17] we now present the results of the comparison between a simulated online adaptive treatment to a clinically implemented CBCT-based plan selection strategy for SCRT and LCRT. The results when comparing plan selection to the online strategy show large and significant reductions for both bowel bag and bladder for all DVH parameters analyzed.

The online strategy results in much less dose to the normal tissue because of smaller margins used. It is likely that this translates into a clinically relevant reduction of toxicity. Toxicity has been mostly reported in prostate patients that are treated with higher doses than rectal cancer. Holyoake et al. [33] conducted a meta-analysis looking at the mean difference in volume for small bowel for different dose levels between grades 0–2 and grade 3 toxicity and a toxicity risk for V10Gy and V40Gy received by normal fractionated radiotherapy. In all included studies the patients were treated with chemotherapy concomitantly. They found evidence for a significant dose-volume-toxicity response effect for a wide range of clinically-relevant doses in the treatment of rectal cancer. Comparison with our results is hampered by the different delineation of small bowel (bowel loops versus bowel bag). For late toxicity for bladder the significance of reduced dose is much less evident. Fiorini et al. [34] summarizes that only high doses (> 60-65Gy) to small volumes and 50-60Gy to whole bladder increases the risk of moderate to high toxicity, analyzed for different treatment sites. These dose-volume-toxicity response fall outside of the clinically-relevant doses for rectal cancer. Harsolia et al. [35] however suggest to limit the bladder wall V30Gy to < 30 cm^3^ (if wall information is not available to use solid V30Gy) based on a large prospective study on prostate patients assessing predictors for grade 2 and 3 chronic urinary toxicity. For acute toxicity for bladder in rectal patients Appelt et al. [36] report a dose-cut-off model of V35Gy and later suggest constraints [37] for bladder of V21Gy < 15% and V25Gy < 5% (SCRT) and V35GY < 22% and V50Gy < 7% (LCRT) in early stage rectal cancer.

This study used a 3 mm margin around the entire CTV volume for the online adaptive treatment. This 3 mm margin is often suggested in the literature for different sites [38–41] when using online MRI image guidance. Even though in principle all shape variations and rotations are corrected with the online strategy, some uncertainties will remain [42, 43]. Intra fraction motion, i.e. shape change of the rectum during treatment, is not accounted for. This has been assessed by Kleijnen et al. [44]. They observed that 90% of the time motion is below 3.6 mm for the CTV when looking at 1 min time intervals. Their results cannot be translated into margins but they state “plan of the day’ approaches [are] only meaningful if imaging, planning, and delivery can be done in under 18 min. Also, delineation uncertainty has always been a prominent factor contributing to the margin [43, 45, 46]. Conventionally, when designing a plan on a single pre-treatment CT scan this uncertainty is systematic in nature. However, for an adaptive procedure with multiple fractions, with a daily (re-) delineation is repeated (or adjustment), that error can be characterized as random [42]. Data on this random uncertainty, obtained under realistic clinical time constraints, is currently lacking and should be quantified. Previously there have been reports on overestimating accuracy with detrimental effects on local control [47].

A limitation of our study is the use of the original delineation of the lymph node region. An online adaptive workflow will be a balance between complexity and speed. Keeping the original lymph node delineation will speed up the adaptive process. Gwynne reports in a review that pelvic vessels have a relatively stable position in relation to the bony pelvis and a 3 mm margin would be sufficient [48–50]. Although the vessels and with that the lymph nodes are stable Nijkamp et al. [6, 7, 9] suggest to use non uniform margins of a 5–13 mm for presacral space for an offline adaptive workflow. This is not due to variety in lymph node position but because of bowel loops moving in and out of the pre sacral volume. Adapting the lymph node region as well might be necessary when using a 3 mm margin, ands need further research.

A limitation of this study is the moderate number of patients [20] and fractions (300) used for analyses. The significance and large difference between the online strategy and plan selection for all dose levels and for both SCRT and LCRT is, however, convincing. The quantification of the added value of online adaptation could benefit from larger patient numbers.

In our study the initially planned dose was used to evaluate coverage of the target volume and dose to the OARs per fraction instead of dose accumulation. Preferably the dose would be accumulated but deformable image registration, especially in the presence or absence of air, has large limitations [32]. By analyzing the dose per fraction we avoided additional uncertainties that would be introduced by deformable image registration and subsequent dose accumulation. However, an accurate deformable image registration algorithm, taking the complexities of the pelvic region into account, should be preferred whenever that becomes available.

To compare de DVHs per fraction a different option could have been to deform the planning CT to the CBCT and thus account for difference in densities due to anatomical changes (for example, air in rectum). However this method introduces an uncertainty as well. If anything, dose calculation on CBCT would favor the online adaptive treatment, because density changes would be accounted for with daily plan creation. For both approaches the uncertainty applies to both the plan selection and online adaptive treatment with the same magnitude and therefore does not affect our results. Nevertheless, we expect that the effect will be small as compared to the dosimetric effect of using much smaller PTV margins.

Online adaptive strategies require not only accurate and fast contouring and treatment planning but also a reconfiguration of workflows and responsibilities. It may very well result in the need of the presence of radiation oncologist, medical physicist and/or dosimetrist at the treatment machines. Also, timeslots may need to be adjusted as adaptation will take additional time [51, 52].

The margin choice (3 mm) is important for the conclusion of this paper. Moving towards online adaptive treatment for rectal cancer the practicality, accuracy and quality needs to be investigated to be able to calculate appropriate margins. This paper however, gives a first estimate of the potential benefit of online adaptation for rectum and helps in the process of prioritizing treatment sites.

## Conclusion

Radiotherapy with online adaptive re-planning of locally advanced rectal cancer reduces dose to the bladder and small bowel significantly, compared to a clinically implemented plan selection strategy.

## Supplementary information

**Additional file 1.** Distribution of selected margins for all patients and all fractions.

**Additional file 2.** Distribution of selected margins per patient sorted on short (5x5Gy) and long (25x2Gy) treatment schedules.

**Additional file 3.** Patient characteristics.

**Additional file 4.** Margin sets based on anatomy as captured on planning CT. (1a) shows an empty rectum with a set of 25 mm, 15 mm, and 0 mm margins (red) for the upper mesorectum (blue). (1b) shows a full rectum with a set of 15 mm, 0 mm, and − 15 mm anterior margins (red) for the upper mesorectum (blue). Yellow is the lower mesorectum.

**Additional file 5.** Flowchart of study comparing target coverage and dose to the organs at risk (OAR). Structures of upper mesorectum (yellow), lower mesorectum (purple), bladder (light blue) and bowel bag (green) were delineated on Conebeam CT. Elective lymph nodes (blue) and presacral space were rigidly propagated from planning CT to Conebeam CT. PTV in red.

## Data Availability

The datasets generated and/or analyzed during the current study are not publicly available since the participants did not consent in sharing the data with third parties.

## Abbreviations

ART: Adaptive Radiation Therapy
CBCT: Conebeam CT
CT: Computed tomography
CTV: Clinical target volume
DVH: Dose volume histogram
GTV: Gross tumor volume
Gy: Gray
IMRT: Intensity modulated radiation therapy
LCRT: Long course radiation therapy
PTV: Planning target volume
RTT: Radiation therapist
SCRT: Short course radiation therapy
VMAT: Volumetric modulated arc therapy
VxGy: Volume receiving x Gy
