# Supplementary figures and images for: Online adaptive radiotherapy compared to plan selection for rectal cancer: quantifying the benefit
Source: Radiat Oncol. 2020 Jul 9;15:162. doi: 10.1186/s13014-020-01597-1 (PMC7371470; doi:10.1186/s13014-020-01597-1)

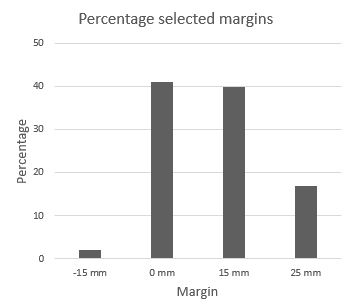

Supplement: Supplementary file 1 — Additional file 1. Distribution of selected margins for all patients and all fractions. [file 13014_2020_1597_MOESM1_ESM.jpg]

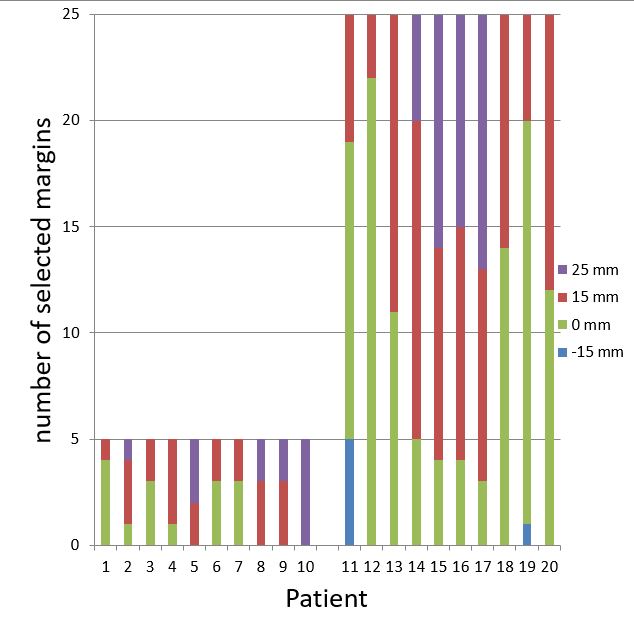

Supplement: Supplementary file 2 — Additional file 2. Distribution of selected margins per patient sorted on short (5x5Gy) and long (25x2Gy) treatment schedules. [file 13014_2020_1597_MOESM2_ESM.jpg]

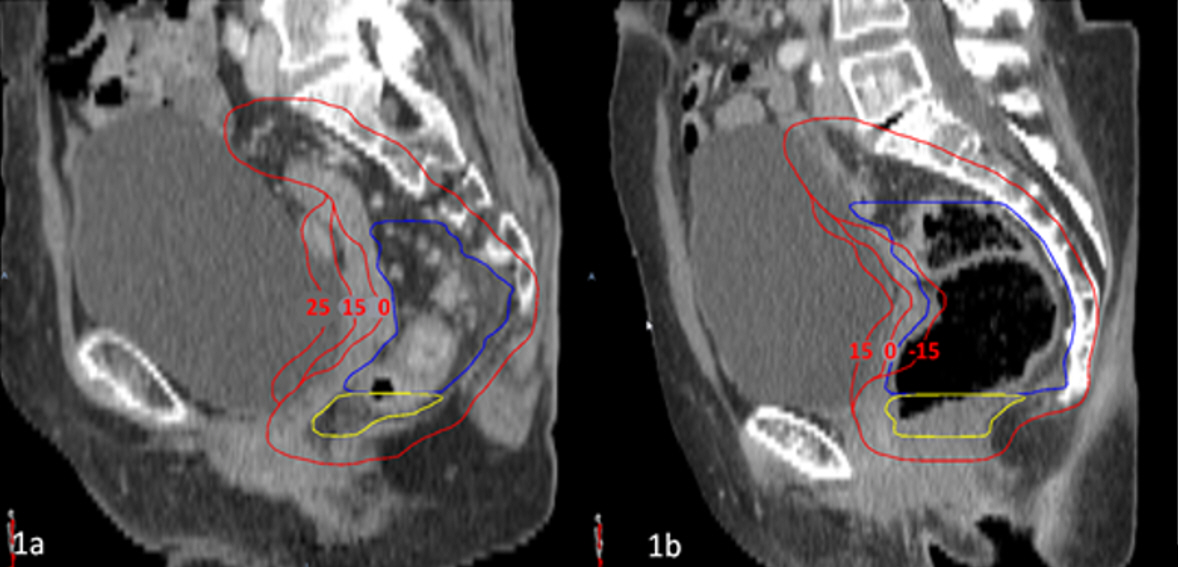

Supplement: Supplementary file 4 — Additional file 4. Margin sets based on anatomy as captured on planning CT. (1a) shows an empty rectum with a set of 25 mm, 15 mm, and 0 mm margins (red) for the upper mesorectum (blue). (1b) shows a full rectum with a set of 15 mm, 0 mm, and − 15 mm anterior margins (red) for the upper mesorectum (blue). Yellow is the lower mesorectum. [file 13014_2020_1597_MOESM4_ESM.jpg]

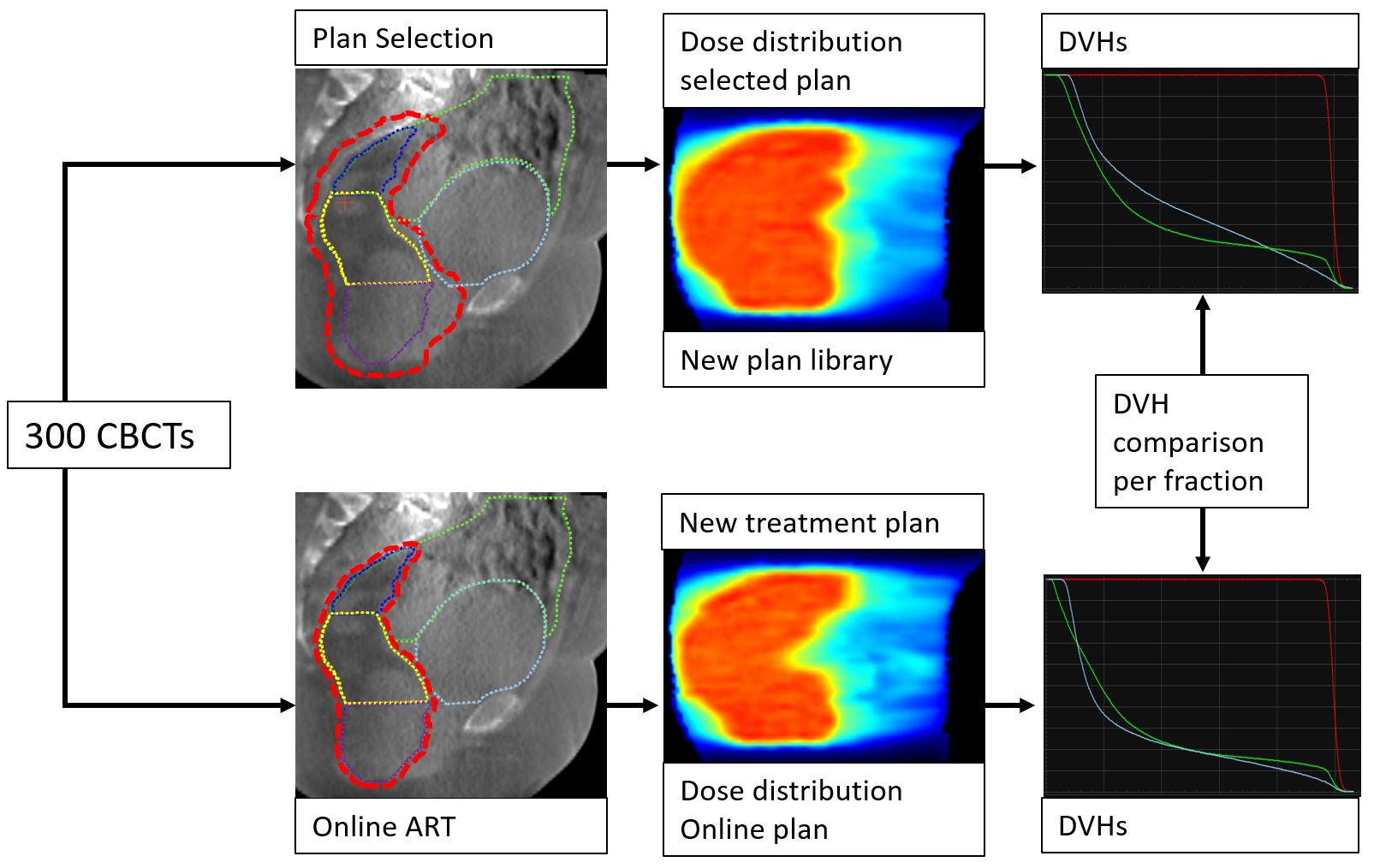

Supplement: Supplementary file 5 — Additional file 5. Flowchart of study comparing target coverage and dose to the organs at risk (OAR). Structures of upper mesorectum (yellow), lower mesorectum (purple), bladder (light blue) and bowel bag (green) were delineated on Conebeam CT. Elective lymph nodes (blue) and presacral space were rigidly propagated from planning CT to Conebeam CT. PTV in red. [file 13014_2020_1597_MOESM5_ESM.jpg]
