# Supplementary material for: Online adaptive radiotherapy compared to plan selection for rectal cancer: quantifying the benefit
Source: Radiat Oncol. 2020 Jul 9;15:162. doi: 10.1186/s13014-020-01597-1 (PMC7371470; doi:10.1186/s13014-020-01597-1)
Supplement: Supplementary file 2 [file 13014_2020_1597_MOESM3_ESM.docx]

Table 1: Patient characteristics sorted on SCRT and LCRT

nr	Age	Sex	Tumor stage	Treatment scheme	Chemo	GTV location	Surgery	Available margins (mm)		
1	65	M	cT3N1M0	5x5 Gy	N	proximal	LAR	-15	0	15
2	70	F	cT3N0M0	5x5 Gy	N	mid	LAR	0	15	25
3	63	M	cT4N1M0	5x5 Gy	Y	distal	ns	-15	0	15
4	71	M	cT3N1M0	5x5 Gy	N	mid	LAR	0	15	25
5	74	M	cT3N0M0	5x5 Gy	N	proximal	LAR	0	15	25
6	56	F	cT3N1M0	5x5 Gy	N	mid	LAR	-15	0	15
7	73	M	cT3N2M1	5x5 Gy	Y	proximal	LAR	0	15	25
8	70	M	cT3N2M0	5x5 Gy	N	distal	APR	0	15	25
9	40	F	cT3N1M0	5x5 Gy	N	mid	LAR	0	15	25
10	78	M	cT4N1M0	5x5 Gy	N	rectosigm	LAR	0	15	25
11	71	F	cT3N1M0	25x2 Gy	Y	proximal	LAR	-15	0	15
12	66	M	cT3N2M0	25x2 Gy	Y	mid	LAR	-15	0	15
13	64	F	cT3N2M0	25x2 Gy	Y	distal	APR	0	15	25
14	65	M	cT3N2M0	25x2 Gy	Y	proximal	LAR	0	15	25
15	55	F	cT4N0M0	25x2 Gy	Y	distal	LAR	0	15	25
16	59	M	cT3N0M0	25x2 Gy	Y	mid	LAR	-15	0	15
17	72	M	cT3N2M0	25x2 Gy	Y	proximal	LAR	0	15	25
18	69	F	cT4N1M0	25x2 Gy	Y	mid	LAR	0	15	25
19	77	M	cT3N2M0	25x2 Gy	Y	distal	ns	0	15	25
20	60	M	cT3N2M0	25x2 Gy	Y	distal	ns	-15	0	15

ns: not specified

LAR: low anterior resection

APR: abdominal perineal resection
